# Foreign body aspiration and mucormycosis: a case report

**DOI:** 10.3389/fmed.2023.1273240

**Published:** 2023-11-06

**Authors:** Hamed Mehdinezhad, Reza Mohseni Ahangar, Mohammad Golparvar Azizi, Mohammad Ghasemian, Zahra Yari, Elham Jafarian, Ali Tavakoli Pirzaman

**Affiliations:** ^1^Department of Pulmonary and Critical Care Medicine, Rouhani Hospital, Babol University of Medical Sciences, Babol, Iran; ^2^Department of Internal Medicine, Rouhani Hospital, Babol University of Medical Sciences, Babol, Iran; ^3^Student Research Committee, Babol University of Medical Sciences, Babol, Iran

**Keywords:** COVID-19, mucormycosis, foreign body aspiration, hemoptysis, rigid bronchoscopy

## Abstract

Over the course of the Coronavirus disease 2019 (COVID-19) pandemic, numerous complications have been documented. In this report, we have detailed an unexpected complication of the severe acute respiratory syndrome coronavirus 2 (SARS-CoV-2) infection that occurred in a 73-year-old female patient who was simultaneously afflicted with mucormycosis and another unanticipated problem. Due to the lack of recovery of the patient after receiving mucormycosis treatment and continued fever, cough and hemoptysis, bronchoscopy was performed for her. During bronchoscopy, we encountered a foreign body that was the cause of the patient’s fever, cough, and hemoptysis. Rigid bronchoscopy was performed and the foreign body was removed from the left main bronchus. The lack of a favorable treatment response after administering antifungal therapy suggested that the presence of a foreign body could potentially act as an underlying nidus, thus influencing the suboptimal therapeutic outcome. Mucormycosis is usually characterized by distinct radiological patterns. However, this case did not present predictable imaging findings, further complicating the diagnostic process associated with this invasive fungal infection.

## 1. Introduction

Since the beginning of Coronavirus disease 2019 (COVID-19) pandemic, the severe acute respiratory syndrome coronavirus 2 (SARS-CoV-2) has resulted in the death of over 6 million people and led to a great variety of complications (1). Patients who have respiratory viral infections are at risk for secondary infections, particularly those caused by bacterial and fungal organisms (2, 3).

An invasive fungal disease known as mucormycosis has a significant mortality rate. The established risk factors for mucormycosis include poorly managed diabetes mellitus, organ transplantation, haematological malignancies, and immunosuppression. A significant number of cases of COVID-19-associated mucormycosis (CAM) were recorded internationally, particularly in India, throughout the second wave of the COVID-19 pandemic (April–June 2021) (4). It is still unknown what caused this CAM epidemic in India. Glucocorticoids, which are used to treat COVID-19, and diabetes mellitus have both been noted as CAM risk factors (5, 6). Alterations in iron metabolism, the severity of COVID-19, and immunological dysfunction brought on by COVID-19 (such as lymphopenia and other symptoms) are some additional aspects that have been hypothesised in the aetiology of CAM (7, 8).

Here, we described a case of SARS-CoV-2 infection who was complicated with both mucormycosis and foreign body aspiration (FBA).

## 2. Case presentation

In April 2022, a 73-year-old woman presented with hemoptysis and was admitted to our hospital. She exhibited symptoms of weakness, chills, fever, cough, decreased consciousness, and dyspnea. The patient had been hospitalized two weeks prior due to severe COVID-19 infection and received treatment with remdesivir and methylprednisolone (40 mg/day for 7 days), resulting in the cessation of her fever, cough, and dyspnea. She was discharged but later experienced mild hemoptysis, along with fever, cough, and dyspnea, leading to readmission to the hospital.

She was a known case of diabetes mellitus (diagnosed since 10 years ago; treated with sitagliptin/metformin 50/1000 mg/day and gliclazide 60 mg/day), hypertension (controlled by medication), ischemic heart disease (managed with coronary artery bypass grafting (CABG) 8 years earlier), and hyperlipidemia (controlled by medication). She had no history of asthma, chronic obstructive pulmonary disease (COPD) or wheeze. She also had no history of smoking.

On physical examinations, the patient was an old lady, lying on bed, ill but not toxic, and answered to the questions. Her blood pressure was 125/80 mmHg; respiratory rate, 22 breaths per min; temperature, 38.2°C; pulse rate, 99 beats per min; and oxygen saturation, 95% (without any oxygen therapy). She had symmetric chest wall movement along with wheezing (detected on left upper lobe). The other examinations were unremarkable. She had elevated lactate dehydrogenase (LDH) (545 U/L) and White Blood Cells (17,000 per μL; neutrophil percentage of 65% and lymphocyte percentage of 33%) at the time of admission (Table 1).

**Table 1:** Laboratory data on admission day.

	Admission day	Normal values
WBC (per μL)	17,000	4,500–11,000
Neutrophil (%)	65	40–60
Lymphocyte (%)	33	20–40
Hb (g/dL)	10.7	Female: 12.1 to 15.1
MCV (fL)	77.6	80–100
PLT (per μL)	342,000	150,000–400,000
LDH (U/L)	545	105–333
CRP (mg/dL)	3.0	< 0.3
ESR (mm/h)	35	Female over 50 years old: < 30
Bilirubin (Total) (mg/dL)	0.5	0.1–1.2
Bilirubin (Direct) (mg/dL)	0.2	< 0.3
AST (U/L)	15	8–33
ALT (U/L)	12	4–36
ALP (U/L)	230	44–147
Troponin	Negative	Negative
BUN (mg/dL)	13	6–20
Creatinine (mg/dL)	1	0.8–1.2
PT (sec)	12	11–13.5
PTT (sec)	25	25–35
INR	1	0.8–1.1
HbA1c (%)^*^	10	< 5.7

WBC, white blood cells; Hb, hemoglobin, PLT, platelets; LDH, lactate dehydrogenase; CRP, C-reactive protein; ESR, erythrocyte sedimentation rate; AST, aspartate transaminase; ALT, alanine transaminase; ALP, alkaline phosphatase; BUN, blood urea nitrogen; PT, prothrombin time; PTT, partial thromboplastin time; INR, international normalized ratio; MCV, mean corpuscular volume. *The most recent HbA1c level.

**Figure 1:**
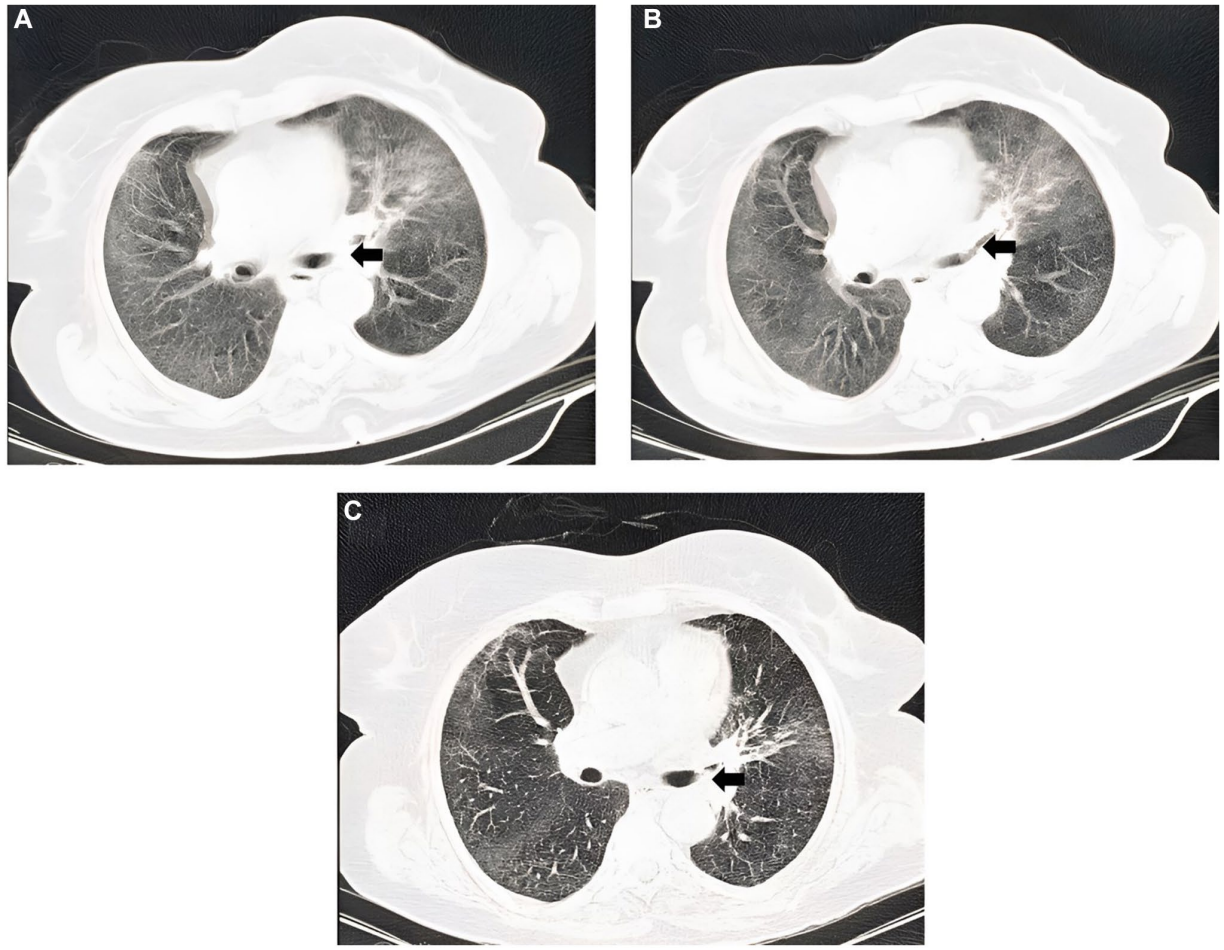
**(A,B)** On the second admission CT scan, there were diffused bilateral ground glass opacities, predominantly in the upper zone along with increased haziness. The narrowing of the orifice of the left upper lobe (leftward arrow) and the air-bronchogram which was behind it were also observed in the left lung. **(C)** On the CT scan before re-bronchoscopy, the orifice of the upper lobe of the left lung had still irregularity (leftward arrow); however, the ground glass opacities and haziness were relatively improved. The ground glass opacities had progressed to linear opacity and linear fibrosis.

Despite a negative result for the COVID-19 polymerase chain reaction (PCR) test, the patient’s computed tomography (CT) scan revealed the presence of lesions such as bilateral ground glass opacities and narrowing of the left upper lobe orifice (Figures 1A,B). As a result, we suspected hospital-acquired pneumonia and treated the patient with levofloxacin. Nevertheless, due to persistent fever, hemoptysis, and pulmonary lesions after 3 days, we suspected coinfection with fungal agents and flexible bronchoscopy was performed for the patient.

**Figure 2:**
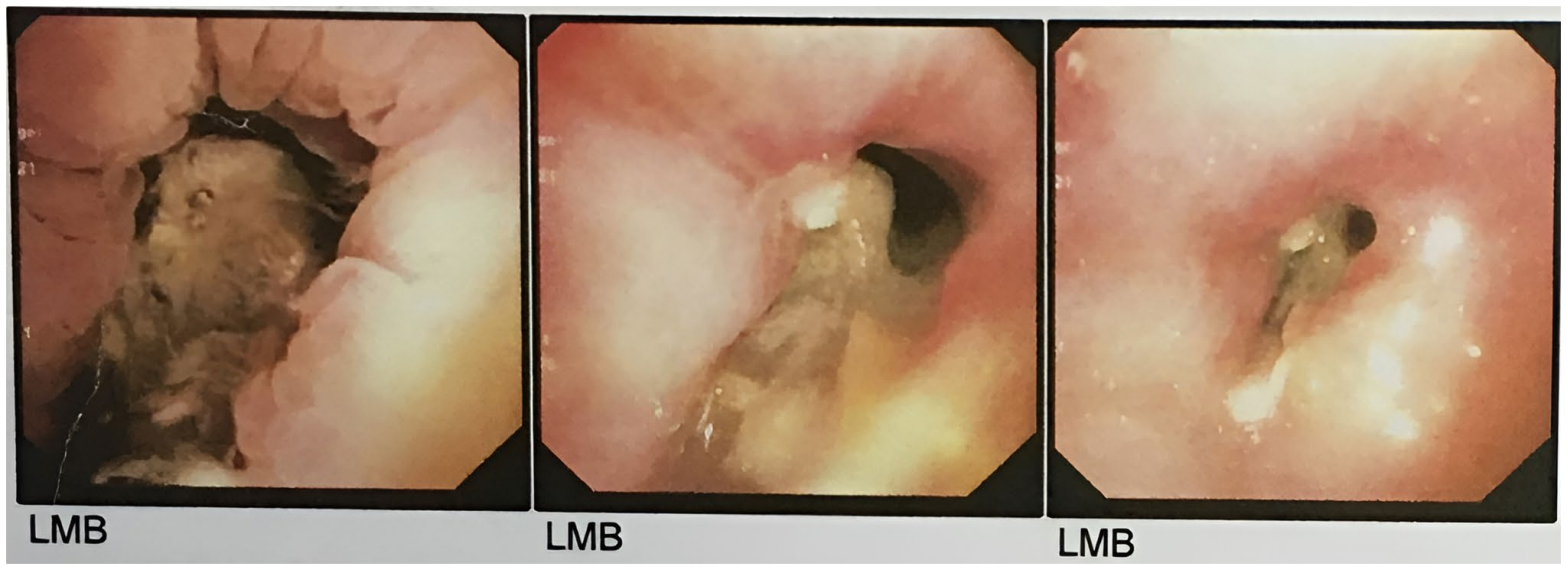
The first round of flexible bronchoscopy showed mucosal irregularity, erythema, and edema, along with dark and dirty ulcerative endo-bronchial lesion at the end of left main bronchus.

**Figure 3:**
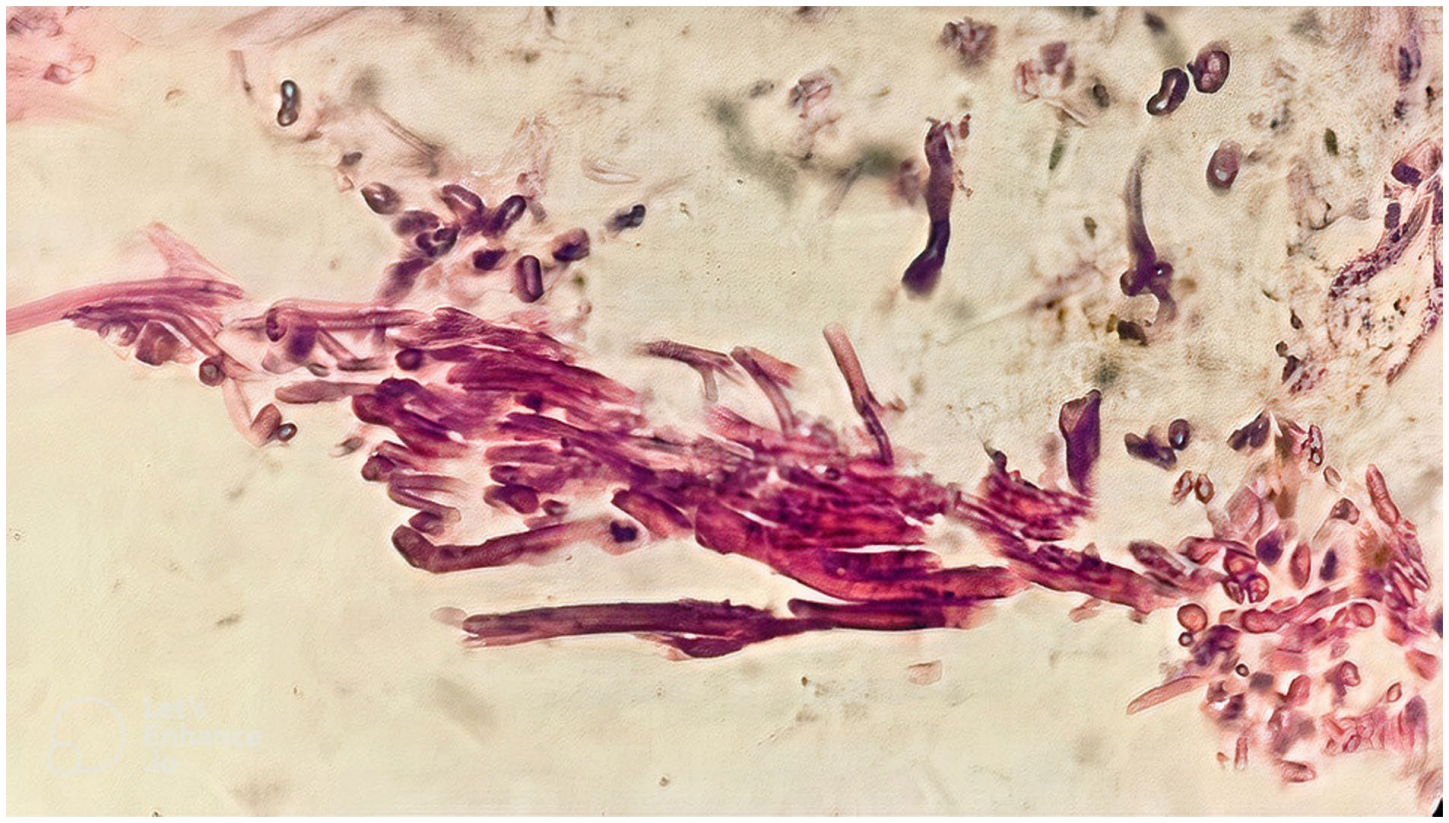
The pathology report, following the first round of flexible bronchoscopy, revealed presence of nonseptated and broad based fungal hyphae consistent with mucormycosis.

Mucosal irregularity, erythema, and edema, along with dark and dirty ulcerative endo-bronchial lesion were observed at the end of left main bronchus (Figure 2), through which the scope could not pass. Multiple biopsies (from the margins of the lesion) and bronchoalveolar lavage (BAL) were provided for further investigation. The pathology report revealed presence of nonseptated and broad based fungal hyphae consistent with mucormycosis (Figure 3). Cytology analysis showed an acute inflammatory process, which was negative for malignant cells.

According to pathology report, mucormycosis treatment was started with intravenous liposomal amphotericin B at a daily dosage of 5 mg/kg. Liposomal amphotericin B was administered for 14 days and the patient was discharged with well general condition and the recommendation of taking itraconazole (200 mg orally twice a day). Paranasal sinus (PNS) CT scan showed no involvement of oral cavity and sinuses with mucormycosis (Supplementary Figure S1).

The persistent clinical symptoms observed in the patient included hemoptysis, as well as weakness and fatigue. Additionally, the CT scan revealed the persistent narrowing of the left upper lobe orifice (Figure 1C). Therefore, another flexible bronchoscopy was performed. Multiple biopsies were performed from the depth of the lesion in the left main bronchus, and two small objects (with dimensions of 0.5 × 0.5 cm, hard density, and cream color) (Supplementary Figure S2A) were accidentally removed.

We determined that rigid bronchoscopy was the best option after taking into account the patient’s overall health, her history of hemoptysis, and the foreign body’s undetermined size. Moreover, the patient’s airway was congested and edematous, and additional flexible bronchoscopy manipulation of that region was linked to a higher risk of severe bleeding. Rigid bronchoscopy was performed and the result was surprising. A foreign body with dimensions of 2.7 × 1.2 cm was removed from the left main bronchus (Supplementary Figure S2B). In fact, the aforementioned two foreign bodies discovered during flexible bronchoscopy were minute fragments of a larger bone that was removed during rigid bronchoscopy and turned out to be a chicken bone aspirated by the patient.

## 3. Discussion

Mucormycosis, a rare but potentially fatal invasive fungal infection, is brought on by mold fungi of the genus *Mucor*, *Rhizopus*, *Rhizomucor*, and *Absidia*, which are all members of the Mucorales order of the zygomycetes class (9). *Rhizopus oryzae* was the most prevalent kind, accounting for 60% of human cases of mucormycosis and 90% of cases of the rhinocerebral variant (10).

Rhino-orbital mucormycosis was the most prevalent manifestation of the current mucormycosis epidemic caused by COVID-19, whereas COVID-19-associated pulmonary mucormycosis (CAPM) was a rare diagnosis (8, 11). Although isolated tracheal or bronchial mucormycosis is infrequent, bronchoscopic abnormalities in CAPM and pulmonary mucormycosis are prevalent (12). All kinds of mucormycosis are most frequently caused by poorly managed diabetes mellitus (13). Damaraju et al. showed that those with diabetes mellitus were more likely to experience Isolated tracheobronchial mucormycosis (ITBM) (79.2%). Additionally, they discovered a few special risk factors, including previous tracheal or bronchial surgeries (such as the location of the anastomosis in a lung transplant) and previous respiratory virus infections (14). It is possible that local factors can change the conditions in the tracheal mucosa of individuals with underlying health conditions (such as uncontrolled diabetes, smoking, use of glucocorticoids, and potentially zinc supplementation), which may lead to the development of mucormycosis in the airways (14, 15). This suggests that ITBM could be classified as a unique form of pulmonary mucormycosis.

Here, we describe a case of SARS-CoV-2 infection that was first diagnosed with tracheobronchial mucormycosis and treated accordingly. Then, because of persistent hemoptysis, he underwent further diagnostic work-up and was later diagnosed with FBA.

Differential diagnoses for CAPM imaging include severe COVID-19, various bacterial pneumonia, tuberculosis (TB), and COVID-19-associated pulmonary aspergillosis (CAPA) (16). Invasive mould infections can be distinguished from other pneumonias by the presence of cavitating nodules, a hypodense sign, an air crescent sign, a reversed halo sign (RHS), and a halo sign (17). CAPM more frequently affects diabetic people after COVID-19 and mimics pulmonary mucormycosis in diabetic patients (11). In order to distinguish between acute COVID-19 and CAPM, it is important to consider the chronology, clinical setting (productive cough, haemoptysis, persistent or new-onset fever, or uncontrolled diabetes), and illness history (11).

Investigations have revealed a wide range of CT results in COVID-19. However, all investigations show that the presence of ground glass opacities, often with a peripheral and subpleural distribution, is the key CT characteristic of COVID-19 pneumonia (18). Additionally, bronchiectasis, lung abscess, bronchitis, pneumonitis, or atelectasis are the most common CT findings in FBA cases (19).

At first, the imagings of our patient were consistent with the imaging findings of the previous case of tracheobronchial mucormycosis (14). However, it was then found that the narrowing of the orifice of the left upper lobe was due to foreign body. Importantly, not all imaging findings can be attributed to a foreign body; Especially when the patient received antifungal treatment, a large part of the edema in the area was reduced, which helped us to take a biopsy sample from the lesion itself in the second round of flexible bronchoscopy and find out the presence of a foreign body.

The diagnosis of isolated tracheobronchial mucormycosis is frequently overlooked and delayed. Importantly, before the biopsy result was available, no case documented in the literature was suspected. Some of the early diagnoses taken into consideration based on imaging and bronchoscopic appearance were foreign body, laryngitis, granulomatosis with polyangiitis, bronchial adenoma, TB, and malignancy. Because sputum smears analysis and fungal culture have little sensitivity, early bronchoscopy is beneficial (20). Bronchoscopy also makes it possible to prepare for eventual surgery or other types of therapy. Because the infection of the main airway can result in edema, granulation tissue, pseudomembrane development, submucosal abscess, exophytic growth, necrosis, and finally cartilage loss, prompt diagnosis is essential to improve results. Significant hemoptysis, stridor, respiratory failure, post-obstructive pneumonitis, and distal airway collapse may all be caused by the luminal constriction, bronchial perforation, mediastinal fistula, and pulmonary artery invasion (14).

In general, it is not advised to use itraconazole to treat mucormycosis. Using itraconazole alone or in combination with amphotericin B to treat mucormycosis is really a rare treatment option. Therefore, itraconazole treatment may be administered in the absence of amphotericin B, isavuconazole, and posaconazole (21). Since isavuconazole and posaconazole were not available, we decided to administered itraconazole in combination with liposomal amphotericin B.

Pulmonary mucormycosis outcomes can be enhanced by surgery. Nonetheless, if mucormycosis affects the tracheobronchial tree, surgical intervention is not an option, and endobronchial therapy (bronchoscopic debulking) may be beneficial (22).

During COVID-19 pandemic, there was a significant rise in the number of mucormycosis cases. The WHO and other responsible organizations had issued warnings about this, prompting us to take these risks seriously and prioritize the safety of our patients. We thus began the patient’s therapy as soon as feasible after being satisfied with the report and pathology-based diagnosis. Additionally, it should be highlighted that the patient had diabetes mellitus, which has been linked to a higher risk of developing mucormycosis in diabetics who had recovered from COVID-19 infection (4). Additionally, a mix of clinical, imaging, cultural, and histopathologic data are often used to make the diagnosis (23). Therefore, it should be noted that our study did not utilize the PCR method to verify the presence of the fungus, which may be a limitation. Moreover, During the bronchoscopy, the patient experienced hemoptysis, reduced consciousness, and difficulty breathing. Additionally, she had recently recovered from SARS-CoV-2 infection. Due to the heightened risk of pulmonary hemorrhage, deeper biopsies were not performed. The PCR technique, along with deeper biopsies, could aid in determining if the patient had a standalone mucormycosis infection or if there was colonization on a foreign body or just colonization alone.

It has been previously reported that aspirated foreign body could act as a nidus for Aspergillus colonization (24). Nonetheless, in the present case, it becomes challenging to make a decision about whether the foreign body acted as a colonizer for mucormycosis. Evidence that the patient had tracheobronchial mucormycosis includes the relative improvement in the patient’s condition, the decrease in edema in the lesion area following the antifungal treatment regimen, and the presence of fungus in the biopsy sections taken from the area around the lesion. However, the lack of a favorable response after antifungal therapy suggests that the foreign body could act as an underlying nidus and contribute to the lack of therapeutic efficacy. On the other hand, the lack of deeper biopsies and PCR confirmation does not allow us to definitively comment on whether this foreign body acted as a colonizer.

This study presents intriguing findings concerning mucormycosis, particularly in the absence of identifiable imaging features typically associated with this condition. Notably, the case highlights the discovery of a foreign body as an underlying factor that became evident in subsequent evaluations. Mucormycosis, a rare and potentially life-threatening fungal infection, is usually characterized by distinct radiological patterns. However, this particular case deviates from the expected imaging findings, adding to the diagnostic challenges associated with this invasive fungal disease. The eventual identification of an underlying foreign body further underscores the importance of a comprehensive and thorough diagnostic approach, as it can potentially unmask hidden contributing factors and aid in the appropriate management of mucormycosis cases.

## 4. Conclusion

Here, we described the first case of COVID-19, who experienced FBA along with mucormycosis co-infection. The absence of a positive treatment response following antifungal therapy indicated that the foreign body may serve as an underlying nidus, potentially contributing to the suboptimal therapeutic outcome. Mucormycosis is usually characterized by distinct radiological patterns. However, this particular case deviates from the expected imaging findings, adding to the diagnostic challenges associated with this invasive fungal disease.

## Data availability statement

The original contributions presented in the study are included in the article/Supplementary material, further inquiries can be directed to the corresponding author.

## Ethics statement

Written informed consent was obtained from the individual(s) for the publication of any potentially identifiable images or data included in this article.

## Author contributions

HM: Investigation, Supervision, Writing – review & editing, Conceptualization. RMA: Conceptualization, Investigation, Supervision, Writing – review & editing. MGA: Investigation, Writing – original draft. MG: Investigation, Writing – original draft. ZY: Investigation, Writing – original draft. EJ: Investigation, Writing – original draft. ATP: Investigation, Supervision, Writing – original draft, Writing – review & editing.

## Supplementary material

Supplementary FIGURE S1 **(A,B)** The Paranasal sinus (PNS) CT scan did not reveal any signs of mucormycosis affecting the mouth or sinuses.

Supplementary FIGURE S2 **(A)** One of the two foreign bodies, removed by the second flexible bronchoscopy. It had dimensions of 0.5 x 0.5 cm, hard density, and cream color. **(B)** The foreign body (Chicken bone) with dimensions of 2.7 x 1.2 cm, removed from the left main bronchus by rigid bronchoscopy.
