# Supplementary figures and images for: Foreign body aspiration and mucormycosis: a case report
Source: Front Med (Lausanne). 2023 Nov 6;10:1273240. doi: 10.3389/fmed.2023.1273240 (PMC10658733; doi:10.3389/fmed.2023.1273240)

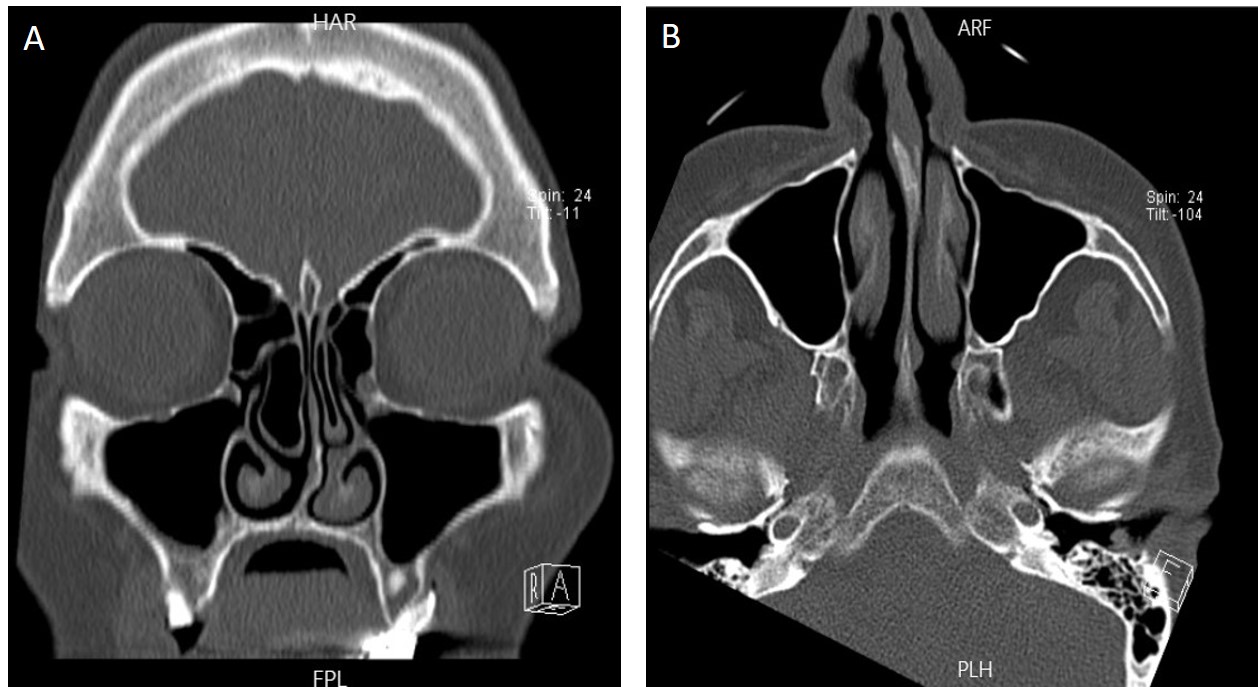

Supplement: Supplementary FIGURE S1 — (A,B) The Paranasal sinus (PNS) CT scan did not reveal any signs of mucormycosis affecting the mouth or sinuses. [file Image_1.JPEG]

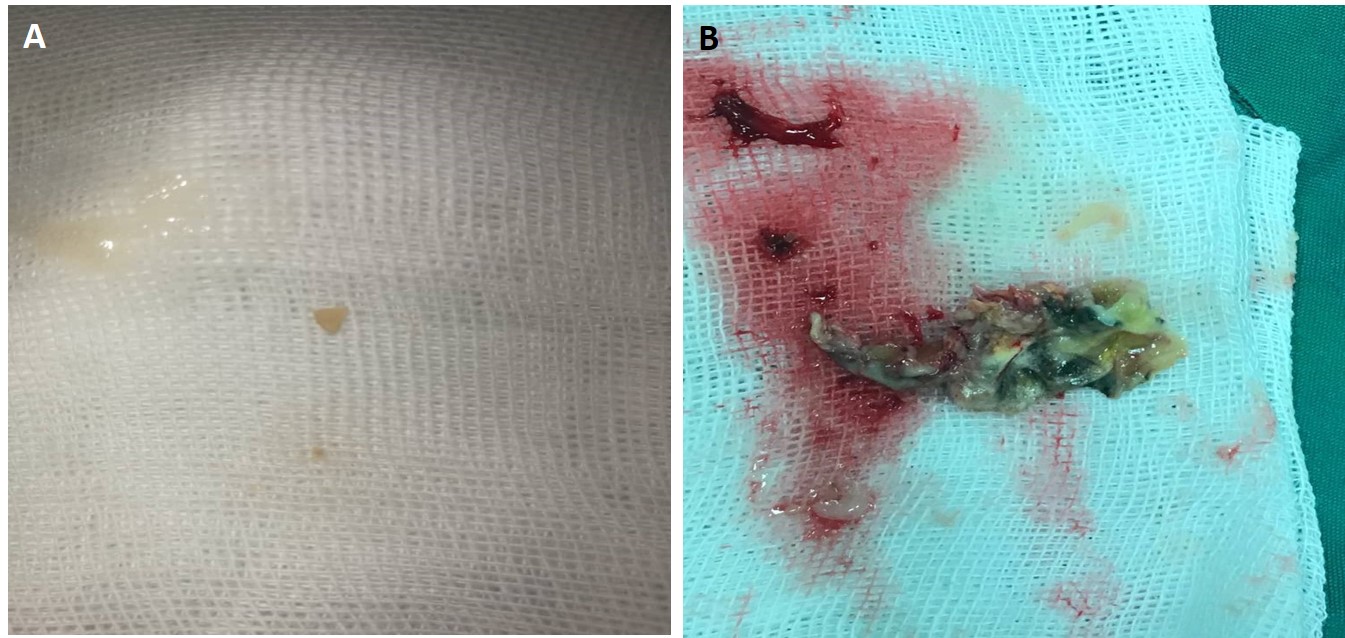

Supplement: Supplementary FIGURE S2 — (A) One of the two foreign bodies, removed by the second flexible bronchoscopy. It had dimensions of 0.5 x 0.5 cm, hard density, and cream color. (B) The foreign body (Chicken bone) with dimensions of 2.7 x 1.2 cm, removed from the left main bronchus by rigid bronchoscopy. [file Image_2.JPEG]
